# Two-eyed seeing as a philosophy to facilitate communication between indigenous counselors and psychiatry about mind and mental health

**DOI:** 10.1192/j.eurpsy.2021.303

**Published:** 2021-08-13

**Authors:** B. Mainguy, L. Mehl-Madrona

**Affiliations:** 1 Education Division, Coyote Institute - Canada, Ottawa, Canada; 2 Medical Arts And Humanities Program, University of Maine, Orono, United States of America

**Keywords:** Indigenous people, two-eyed seeing, explanatory pluralism, cross-cultural communication

## Abstract

**Introduction:**

The term “two-eyed seeing” is spreading across North America as a concept for explanatory pluralism. The concept was brought into academic science by Albert Marshall, a M’iqmaq from Nova, Scotia, Canada. It speaks to the idea that indigenous knowledge is an equally valid way of conceptualizing a phenomenon as is contemporary science. Marshall’s famous example compares a traditional M’iqmaq story about the origins of the large tides in the Bay of Fundy with contemporary oceanographic geology findings and simulations.

**Objectives:**

We wanted to explore how this two-eyed seeing model could be applied to mental health to facilitate a dialogue between psychiatry and traditional cultural healers.

**Methods:**

We reviewed the existing literature on two-eyed seeing within mental health care using PubMed, IndexMedicus, OneSearch, and Google Scholar. We presented a course on two-eyed seeing for indigenous mental health services and two-eyed seeing for addressing trauma in indigenous communities and surveyed the participants about the two-eyed seeing concept. We offered this course primarily to providers within indigenous communities and also for other interested counsellors.

**Results:**

Participants in our trainings were enthusiastic about the role of two-eyed seeing for improving communication among indigenous providers and patients and non-indigenous providers. Most indigenous counselors had not heard of two-eyed seeing and were quite enthusiastic about its affirming nature and how it gave them a basis for dialogue with non-indigenous practitioners.

**Conclusions:**

Two-eyed seeing allows a rich dialogue between European-derived practitioners and indigenous people that enabls each to appreciate the other’s perspectives, leading to greater cooperation and collaborative treatment.

**Disclosure:**

No significant relationships.

